# Large-scale carbon and nitrogen isotopic investigations into caprine diet and management at Tepe Yahya, southeastern Iran

**DOI:** 10.1371/journal.pone.0332662

**Published:** 2025-09-25

**Authors:** Melina Seabrook, Jesse Wolfhagen, Suzanne E. Pilaar Birch

**Affiliations:** 1 Department of Anthropology Harvard University, Cambridge, Massachusetts, United States of America; 2 Department of Anthropology and Department of Geography, University of Georgia, Athens, Georgia, United States of America; University of Padova: Universita degli Studi di Padova, ITALY

## Abstract

Tepe Yahya, a small urban center in southeastern Iran, was embedded in cross-cultural exchange with the many entities of southwestern Asia. Besides an understanding that caprines dominated the animal economy, we have little knowledge of how these animals were managed. The site was discontinuously occupied from the Neolithic to Sassanian periods (6500 BCE to 200 CE). As one of the longest occupied archaeological sites in Iran, Tepe Yahya provides a unique opportunity to examine whether diversity in caprine management accompanied the site’s development and expanded need for provisioning. We examine the diet of 254 sheep and goats between 6500 and 2000 BCE, the Neolithic to Bronze Age, using carbon (*δ*^13^C) and nitrogen (*δ*^15^N) stable isotopic analysis, and estimate the percentage of C_3_ plants in animal diets using a custom isotopic mixing model developed in *R* with “simmr.” While isotopic averages indicate diet was consistent through time, dietary outliers demonstrate the simultaneous presence of multiple management strategies, with certain animals receiving limited access to the full variety of plants available in the landscape. Goats and sheep show statistically significant differences in the percentage of C_3_ plants in their diets, with goats always eating higher portions of C_3_ plants than sheep. These differences are likely a result of human interference rather than substantial environmental changes, which are not reflected in the isotopic values. Increasing isotopic variability over time points to a broader array of caprine management strategies, through greater environmental use or more discrete herds, as well as the introduction of nitrogen-fixing plants into select animal diets. The Tepe Yahya caprines continue to reveal the multifaceted nature of managerial strategies and their relation to urban centers.

## Introduction

Archaeofaunal collections are hidden gems in museum storerooms. These ecofacts are the waste remains of various economic and cultural practices [1]. Excavations in the 19^th^ and early 20^th^ century rarely prioritized the collection of animal bones, assuming the reliance on domesticates such as sheep, goats, and cattle. When excavators collected faunal material, they were sent to museums with their fellow artifacts. Over the years, these legacy faunal collections have had only a few projects, and were otherwise deprioritized, overlooked, or even forgotten. Returning to these collections can make previously unrecognized contributions to our understanding of the past [1].

### Revitalized return to museum collections

Museums and their collections face various challenges worldwide. Budget cuts, reduction of staff and facilities, as well as accidental and targeted destruction threaten collections [2–4]. There is an ongoing curatorial crisis with which archaeologists and curators continue to grapple [5,6]. The rippling effects of COVID-19 led to the cessation of several years of archaeological excavations, rethinking research projects for students, and the need of museums to adapt to virtual engagement [7]. As will be demonstrated in this study, new research on museum collections benefits researchers, museums, and knowledge production.

However, working with museum collections has its own challenges. Museums house thousands of different types of assemblages of varying sizes and conditions [8]. Some assemblages may be inaccessible because of bureaucracy or overwhelmed and understaffed internal systems [9]. Securing permissions can be time-consuming. These challenges can be met by establishing shared goals between curators and researchers can facilitate collaborations that benefit museums, collections, and scholars [10]. Research on museum collections is an investment in the continued curation of materials that maintain cultural and scientific value. Collection work is imperative for minimizing backlog as ongoing excavations continue adding to museum storerooms [11,12]. Returning to legacy collections extends beyond documentation but produces new knowledge [13,14]. A joint project between the Iran National Museum and the Institut Français de Recherche en Iran is documenting many legacy faunal collections to expand our knowledge of human animal relationships on the Iranian Plateau, by revealing the abundance of species utilized, the introduction of domesticated animals and environmental influence on species abundance and subsistence patterns [15]. Continued publication on museum collections demonstrates the necessity of museum maintenance and funding to Boards of Trustees or other granting agencies.

This project focuses the legacy archaeofaunal assemblage from the site of Tepe Yahya in southeastern Iran. Pandemic-related travel restrictions increased the impetus for focusing on material already held in research institutions. Stored in Harvard’s Peabody Museum of Archaeology and Ethnology in Cambridge, Massachusetts, the assemblage consists of over 25,000 bone fragments dating from the Neolithic, c. 6500 BCE, to the Sassanian period, c. 225 CE. Richard Meadow’s 1986 work on the Tepe Yahya fauna documented over 19,000 of these faunal fragments from the Neolithic and Early Bronze Age periods [16]; since that time, the assemblage has been used as teaching material but not rigorously reanalyzed. Returning to a subset of sheep and goats from the Tepe Yahya collection with methods developed and refined during the intervening 40 years, these animals can once again contribute to discussions of caprine husbandry during the formation of urban settlements. This study provides new insights by applying carbon and nitrogen stable isotope analysis to examine caprine diets reflecting both the intentional and unintended outcomes of human-animal interactions in across time at Tepe Yahya.

### Tepe Yahya

Fifty years after the end of the excavations, which occurred between 1969–1975, Tepe Yahya remains a highly regarded site in Iranian Archaeology [17]. The extensively studied pottery corpus continues to be integral in refining the chronology of contemporary sites across southeastern Iran and more broadly in Southwestern Asia [17,18]. Over the course of its occupation, Tepe Yahya grew from a village to a small urban center. The site is 2.5 hectares with an 18-meter high tell at the center [19]. Despite its small size, Tepe Yahya was an enduring regional waypoint with direct ties to the other cultural entities in Southwestern Asia. It sits along a major east-west trade route that connects the societies of southeastern Iran with Baluchistan and the Indus Valley in the east and Mesopotamia in the west [20].

**Fig 1:**
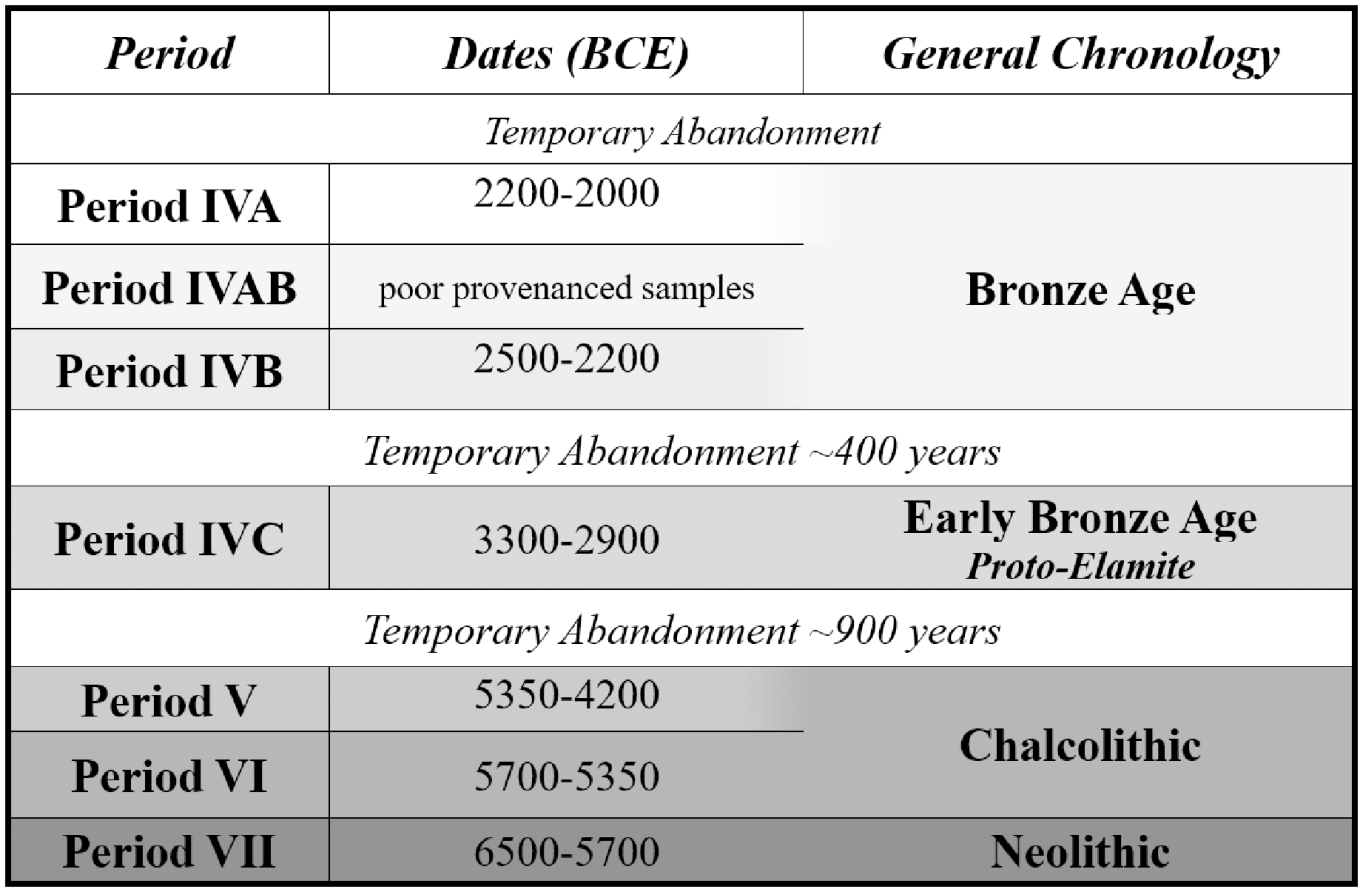
Tepe Yahya Chronology. Tepe Yahya periods discussed in the manuscript, earliest on the bottom. Dates are based on Eskandari et al., [18].

Occupation at Tepe Yahya lasted over 6,000 years, from 6500 BCE to 225 CE, but was not continuous. The longevity of settlement allows for investigations into potential diachronic change in management strategies. Of particular interest are the major ‘transitional’ phases between the Neolithic and Bronze Ages (Fig 1) [21]. Two several-century abandonments occurred, separating the Neolithic and Chalcolithic from the Early Bronze, corresponding here to the Proto-Elamite occupation, and this period from the Middle Bronze Age. The episodes of abandonment create discrete temporal boundaries creating a framework for diachronic comparisons.

#### Cultural interactions at Tepe Yahya.

Tepe Yahya was first established as a village around 6500 BCE. Bevel rim bowls, a pottery style with long-established ties to the Uruk Culture from Mesopotamia, are present in Period V [22]. Lapis lazuli and obsidian objects are also more abundant by this period [19].

The Early Bronze Age occupation at Tepe Yahya corresponds with the arrival of people thought to come from the Zagros who brought Proto-Elamite administrative systems and pottery styles [23]. Tepe Yahya is one of the easternmost example of the Proto-Elamite occupation, corresponding to the Early Bronze Age, with two dozen tablets written in Proto-Elamite script [24,25].

After approximately 400-years of abandonment, people reoccupied Tepe Yahya. There is evidence of a chlorite workshop; vessels from which have been found in Mesopotamia and the Indus Valley, further demonstrating Tepe Yahya’s integral role in regional trade [26]. Other pottery produced at the site also contributes to studies on the development of the endemic Halil-Rud culture of southeastern Iran and the unique local practices [27,28]. There is evidence of Indus seal impressions from Period IVA, highlighting additional connections further east [22]. Thus, it is clear Tepe Yahya was an enduring place of cultural interactions and regional importance despite its small size [29].

The extensive Tepe Yahya faunal assemblage provides an opportunity to examine broad patterns of animal management across time in a place with many cross-cultural interactions. For many contemporary sites, this is not possible due to a lack of or less faunal material. Caprine management in Southwestern Asia is regionally variable [30]. Understanding animal husbandry in one region does not guarantee a widespread or general understanding of animal husbandry throughout Southwestern Asia. Most animal management studies of Southwest Asia come from the Anatolia, the Levant, Mesopotamia, or Northern Iran [30–34]. This is the opportunity to contribute to the understanding of general patterns of caprine management strategies in a particularly cross-cultural area with no previous work of this magnitude.

Large datasets increase the statistical power of our work. The precision of our estimates increases with more samples, particularly for variables like variability improving our ability to identify real divergences in practice [35]. Capturing more of the existing variation safeguards against skewed interpretations caused by a few individuals in each data set. However, we are still limited in sample sizes for some periods due to differences in faunal abundance, less material overall from earlier periods, and poorer preservation. Though we must deal with inevitability of unequal sample sizes between periods, the ability to make intra-site analyses is a worthwhile first step for exploring the variety of management patterns that occurred.

### Caprine management

Caprines make up the majority of the Tepe Yahya fauna, indicating people’s reliance on these animals [16]. Investigating caprine diets provides a window into human decisions regarding this essential economic component. How were people caring for their animals? Were caprines foddered with domestic crops or allowed to roam in pastures? Each strategy comes with tradeoffs. Foddering animals allows for consistent dietary control and increased security of animals, but necessitates greater agricultural output and labor for direct feeding, as well as, increased vigilance of overall animal health to ensure caprines consume all necessary dietary components [36]. In smaller, less dense settlements, people can easily rear and raise their animals locally [37]. In the early Neolithic periods at Tepe Yahya, there was evidence of rooms for penning animals, suggesting that animals were being kept and herded locally [19]. However, as urbanism increases, more people cluster together, leaving less room for animal herds. Pasturing animals feed themselves, requiring less direct effort from people. However, there is greater need for environmental vigilance, moving animals across the landscape for better resources when pastures are exhausted [38]. More movement across rugged landscape may leave animals more vulnerable to predators and the elements [39]. While the animals themselves were pushed out, urban centers are the main places of consumption, with growing numbers of people to feed [40]. The number of people originating outside the valley seems to have increased over time, with more people living at Tepe Yahya in later periods [19]. Thus, broader variation in the diets of caprines because they would need to travel greater distances across more varied landscapes to provision the urban population [41].

### Urban provisioning

Urbanization and the accompanying diversification and specialization of labor created multiple specialized economies [42,43]. Urban centers had to rely on rural hinterlands to provision inhabitants. Caprines were desirable in urban centers for both meat consumption and their secondary products, milk, hair, wool, dung, which served many of the specialized, urban industries [34,44]. Procuring the caprines from the hinterlands was a dynamic process, dependent on many factors such as environmental constraints, and herd health, while balancing the desires and priorities of rural provisioners and urban consumers [44]. The caprines we observe in the faunal assemblages at Tepe Yahya reflect multiple levels of decision making from different actors. Further investigation of the caprines through other avenues will expand our ability to understand the many variables that influence urban provisioning.

At Tal-e Malyan, an ancient city in the central Zagros occupied between 3400 BCE and 1000 BCE, Melinda Zeder found differences in caprine procurement patterns across time and place within the expansive, 135 hectare site [43,45]. During the peak urban phase (2400−1800 BCE), urban inhabitants procured meat primarily through indirect channels, limiting the variation observed in species and type of meat cuts. Zeder notes the ages of the animals are slightly more variable than the preceding period; however, the ratio of sheep and to goats was most even, suggesting animals coming from more local herds than from pastoral nomads [43]. The Tal-e Malyan pattern already contrasts with what we know of the Tepe Yayha caprines, where goats always outnumber sheep at least two to one and the age at time of death is highly variable [46]. Thus, within the Zagros mountains, many strategies of urban provisioning took place and more data is needed to understand the mechanisms.

### Mobile pastoralism

Another facet of caprine management as urbanism develops is the relationship between urban centers and pastoralist communities. Mobile pastoralism is an economy characterized by reliance on caprines and their products. People maintain larger herds of animals, and ensure proper pasturing of the animals, utilizing larger portions of the landscape through seasonal movement [47]. The adaptability of mobile pastoralism to grow, shrink, or change form in the face of hardships made it a resilient way of life; however, it also made it difficult to study archaeologically. Stable isotopic analysis may provide a window into mobile pastoral provisioning of urban centers like Tepe Yahya.

### Dietary investigations using stable isotopic analysis

The use of carbon and nitrogen stable isotopes for dietary and environmental investigations in archaeology is well established cf. [41,48,49]. The stable isotopic analysis of sheep and goats can contribute to our knowledge of animal management via feeding practices and the paleoenvironment cf. [48,50,51]. Bone collagen is slow to turnover; therefore, the values measured from this substrate can provide insights into the animals’ average diet over several years of adult life [52,53]. Isotopic measurements from animals under 12 months of age likely reflect the dietary influence of the mother [54]. Young adult animals’ isotope values most accurately reflect diet in a shorter period. Older animals’ values will be more homogenized, and periods of differential feeding will be obscured [53].

#### Carbon.

Carbon stable isotope ratios (δ^13^C) in animal tissues are derived from differential plant consumption. Plants fix carbon from carbon dioxide during photosynthesis. Different photosynthetic pathways cause fractionation effects by discriminating against the heavier stable carbon isotope, ^13^C [55]. C_3_ plants strongly discriminate against ^13^C during photosynthesis, resulting in more negative 𝛿^13^C values than C_4_ plants, which have higher 𝛿^13^C values [56,57]. Plant carbon is metabolized and incorporated into the tissues of animals [58]. Therefore, δ^13^C values measured from animal tissues reflect dietary proportions of C_3_ or C_4_ plants. Animals with 𝛿^13^C values below −20‰ have a primarily C_3_ diet, and animals with 𝛿^13^C values above −9‰ have a primarily C_4_ diet. Those with intermediate values have a mixed C_3_/C_4_ diet.

The variety of isotopic values in the herd can be attributed to several factors. Elemental isotopes undergo different fractionation processes that are shifted by climatic variation, plant parts, and animal physiology [59]. Water stress, high temperatures, and generally environmental aridity can enrich the δ^13^C values of C_3_ plants by 2–3‰ [60,61]. The resulting δ^13^C values in animals would be less negative, giving the impression of a more substantial C_4_ input. Salinity also decreases the level of ^13^C discrimination in the plants, increasing the δ^13^C values. Domestic crops in improperly maintained fields may experience this effect, again resulting in relative less negative animal δ^13^C values [62].

#### Nitrogen.

Stable nitrogen isotope ratios (δ^15^N) in animal tissues primarily reflect dietary protein consumption and are often used to assess trophic position. Nitrogen moves through the atmosphere and into soils, where bacteria fix it into organic forms that are then incorporated into plant and animal tissues [63]. Soil composition and soil depth can influence the nitrogen uptake of plants [64]. Different plant parts, roots vs stems vs leaves, require different amounts of nitrogen and can display internally distinct δ^15^N values between the different tissues [65]. Mammals generally have a δ^15^N enrichment of 3‰ above their diet [58,66]. This pattern suggests herbivores should be more enriched in ^15^N than the plants they consume, and carnivores more enriched in ^15^N than herbivores.

While the stepwise trophic relationship of δ^15^N is relatively consistent, the δ^15^N within a given set of animals, such as caprines, can vary considerably. Nitrogen is a necessary nutrient for healthy soils in agricultural crop production [67]. In more arid environments the lighter isotope of nitrogen, ^14^N is reincorporated into soils at higher rates and leaving the heavier isotopes, ^15^N, in plant tissues, enriching them [68]. Tissues of young, un-weaned, and recently weaned animals are more enriched in ^15^N from consuming their mother’s milk [69]. Fertilizing plants with manure can enrich δ^15^N values by increasing the soil’s bioavailable nitrogen [70,71]. Domestic animals eating fertilized plants are found to have higher δ^15^N values than wild counterparts without access to fertilized crops [72].

### Environment and paleoclimate

**Fig 2:**
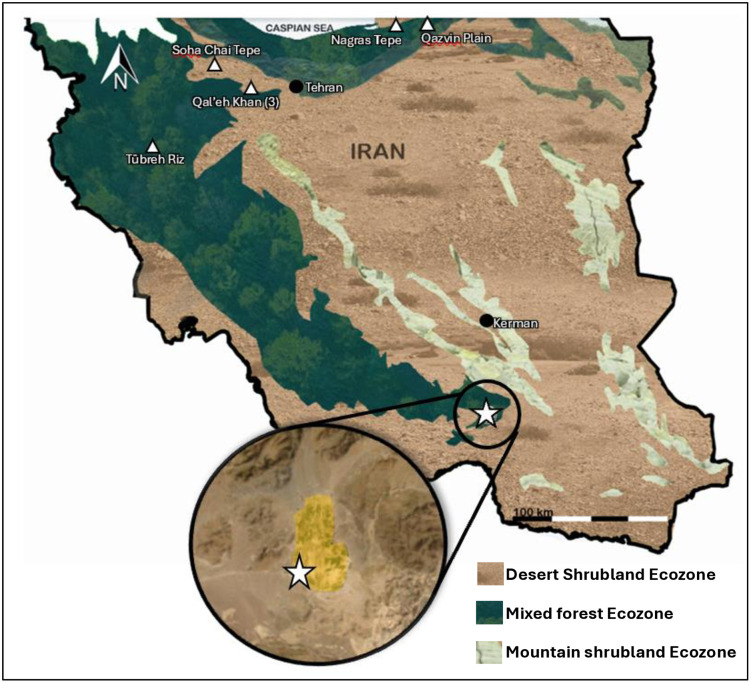
Map of Iran Ecozones. Inset with the arable cropland of the Sogun Valley highlighted in yellow. Tepe Yahya is marked by the white star. Black circles: Modern cities, White triangles: archaeological sites with caprine isotopic information. Map made in R and Photoshop, ecozones modeled after E**coregions 2017 (*CC-BY* 4.0*).* Inset map from USGS Earth Explorer (Public Domain), and photoshop.

The southern Zagros of southeastern Iran hosts a convergence of three main biomes: the end of mixed forests, the coastal desert shrubland and arid montane shrubland (Fig 2). Although Tepe Yahya lies within the forest biome, at 1500 meters above sea level it is on the edge of the juniper and pistachio forest at the higher elevations, 2000 m.a.s.l and up [16]. The overall vegetation cover in the immediate region is sparse today. The paleoclimate was influenced by the Mediterranean Winter Rains, Indian Summer Monsoons, and the InterTropical Convergence Zone (ITCZ) [73]. Over the several thousand years of Tepe Yahya’s occupation, the region experienced multiple dry, cooling events between warmer, humid times [74]. There was more precipitation in the Early Holocene due to the increased strength of the monsoon cycle [75]. Around 7,500 BCE, the influence of the Indian Summer Monsoons decreased and combined with changing solar insolation, led to the drier climate that persist today [75]. However, these general observed patterns may not accurately reflect microclimatic variation in the high mountain valley of Tepe Yahya.

**Fig 3:**
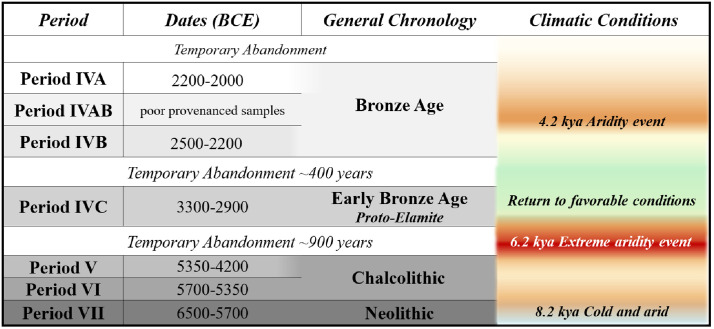
Tepe Yahya chronology and climate. Climatic conditions discussed in the text visualized on the right side of chronological table.

Occupation and abandonment at Tepe Yahya do not appear to correlate with the macroclimatic variation in southeastern Iran. Paleoclimatic data comes from sediment cores of lake deposits across Iran [76]. Settlement at Tepe Yahya began as the climate oscillated between the cold and warm dry periods in the Middle Holocene [77]. The site remained occupied through a significant climatic event at 8.2 kya (6,200 BCE), which was known for its colder and arid conditions (Fig 3) [74]. The lengthy abandonment of Tepe Yahya after Period V, c. 4200 BCE, broadly corresponds to the 6.2 ka event characterized by extreme aridity event and long-lasting droughts [74]. Period IVC, 3300−2900 BCE, overlapped with increased precipitation, creating favorable conditions for the region [75]. However, the temporary abandonment of Yahya after Period IVC, c. 2900 BCE, also occurred during these favorable conditions. When settlement at Tepe Yahya restarted in Period IVB, around 2500 BCE, precipitation had waned, leaving a dry and warm climate [75]. The Bronze Age occupation at Yahya lasted through the start of the 4.2 Ka aridity event (2200 BCE) across Southwestern Asia, long thought to have contributed to the downfall of the Akkadian Empire cf. [78]. The subsequent abandonment of the site, around 2000 BCE, may have been related to the continued aridity of the area [76].

### Plants in the environment

Iran has one of the world’s highest diversities of plant species [79]. The sheep and goats of Tepe Yahya would have had access to a wide range of C_4_ and C_3_ plants on the landscape. The Sogun Valley in which Tepe Yahya lies supports rain fed crop land, and in some parts even more herbaceous ground cover (see inset, Fig 3). However, outside of the valley the vegetation is sparse and minimal. Our understanding of the immediate region’s variety of plants and land cover is incomplete. The wild vegetation of the Sogun Valley of Tepe Yahya was not thoroughly documented by excavators [80]. Pollen records from the excavation indicate *Chenopodium*, mostly C_3_ plants, are the most abundant [16]. The pollen record also indicates the presence of oaks, junipers, and legume species in various quantities [16]. Yet, the pollen record is an incomplete proxy. Pollen abundance and distribution are variable between plant species and pollination type. Almond trees and legumes, for example, produce low amounts of pollen distributed by insects, in contrast to Pistachio, which is wind pollinated [80]. All three are poorly represented in the Tepe Yahya pollen record yet would have been present on the landscape [16]. Soil disturbances such as animal burrowing can contaminate the pollen record for a given period [16]. Wheat is present in all periods at Tepe Yahya, and barley is present from Period VI forward, indicating substantial agricultural production [16]. Oat was also cultivated. Other wild C_3_ plants, such as Prosopis, must have also occupied the landscape [80].

C_4_ plants are specialized for arid environments [81]. Iran has high percentages, anywhere between 10% and 50% depending on the region, of C_4_ eudicots such as *Aristida abnormis,* a widespread desert adapted shrub [79]. The southern Zagros is unique in that C_4_ plants are more common at higher altitudes as they are better adapted for the harsher, more arid conditions of summer compared to their C_3_ counterparts [79,82,83]. C_4_ grasses are also more abundant in the southern Zagros than in other parts of Iran [84]. The immediate vicinity of Tepe Yahya likely hosted around 25–50% C_4_ shrubs, grasses, and sedges. *Salsola tragus*, a thistle, pollen is present in small quantities in pollen records [16]. Sedge pollen likely comes from C_4_ varieties, such as *Cyperus*; however, no differentiation was made previously [16,79].

#### Millet.

The role of millet in caprine diet remains an unknown variable at Tepe Yahya. While millet is a cereal crop, it is a C_4_ plant, meaning animals foddered with millet will have less negative δ^13^C, unlike animals foddered with wheat or barley. Millet has only been recovered at Tepe Yahya in periods IVB-IVA, between 2500 and 2000 BCE [16]. The reliance on dry farming and winter cereals meant that millet, best harvested in late summer, was less crucial for the farmers at Tepe Yahya. Though its presence is undeniably scarce at Tepe Yahya, the domestic context in which it originates may bias our interpretation. If caprines were foddered with millet that was not produced for human consumption, it likely would occur elsewhere on the site [85]. Thus, it would be rare for millet to enter domestic contexts.

## Materials and methods

### Tepe Yahya assemblage

Three hundred fifty-five mandibular fragments, 254 goats and 101 sheep, identified to the genus level using ZooMS were available for collagen extraction [46]. No permits were required for the sampling, which complied with all relevant regulations. Looking at a larger number of animals increases the visibility of potential patterns, allows for the power of statistical analysis, and the potential to observe outliers. Of the 355 caprines, 334 had enough mandibular bone preservation to attempt collagen extraction for carbon and nitrogen isotopic analysis. Ideally, all sample weights fall between 100–500 mg to ensure enough material for duplicate or secondary runs or poor preservation quality. Due to the variable preservation of bone, sample weight ranged from 33 to 1,174 mg. Fragments of bone were used rather than powdered bone, saving time and allowing for identification of the collagen shadow [86].

All samples were prepared in Harvard’s Anthropology Multiuser Laboratory. All reagents were of lab grade. Bone fragments from each mandible were demineralized in 0.5 M HCl until only a collagen shadow remained [87]. The acid was discarded, and the remaining collagen was rinsed in de-ionized water five times. The samples were soaked in sodium hydroxide (NaOH) overnight to remove potential humic acid contamination [88]. After 24 hours, samples were removed from NaOH and rinsed another five times with de-ionized water. Samples were lyophilized until completely dry on a Labconco lyophilizer in Harvard’s Bauer Core. Lyophilized collagen was weighed into tin capsules within five micrograms of the 50-microgram target. Ten percent of samples were run in duplicate to assess internal variation. Samples were run in the Pearson Lab in Harvard’s Geosciences Department on an Elemental Analyzer-Isotope Ratio Mass Spectrometer by lab manager Dr. Susan Carter. Standards included were USGS 40, USGS 41a, Tyrosine, and L-glutamine. The overall coherence of duplicate samples was good, apart from a few samples (S1 File). Given the success of other duplicates, the few incongruent samples were rerun for a third time for comparison. Atomic C:N was calculated using the formula provided by Guiry and Szpak [89].

### Data processing

Here, we consider atomic C:N ratios between 2.9 and 3.6 acceptable [90]. Though upper values between 3.3 and 3.6 have recently been questioned for validity [88], their inclusion in this dataset is merited. Based on Guiry and Szpak’s [88] experimental studies, values above 3.28, the known modern ratio, inherently contain exogenous carbon, likely due to latent humic acids. As stated above, all samples were soaked in 0.1 M NaOH for 24 hours in pretreatment, which should remove most humic acids. This does not preclude the presence of other exogenous carbon but addresses one of the major factors. The authors found that if atomic C:N ratios plotted against carbon values appear correlated, there is likely substantial humic contamination [88]. However, this assumes a narrow range of values. The raw data for this assemblage immediately shows a wide variation in δ^13^C values for both sheep and goats in all periods. Thus, it is unlikely to find any correlations to gauge contamination between the atomic C:N and δ^13^C values [88].

Though there might be potential for contamination in carbon values, humic acid has negligible effects on δ^15^N values [88]. Around half of the outliers in this assemblage are considered outlying because of their nitrogen values, suggesting they are real anomalies and are not created by humic contaminants. Moreover, many outliers have atomic C:N values between 2.9 and 3.2, which is the accepted modern range of collagen for these animals [89]. The values in the higher range of atomic C:N are similar to and correspond well within the samples with ‘true’ atomic C:N values. Thus, including values between 3.3 and 3.6 does not significantly alter the analysis.

### Isotopic mixing model

To understand the wide range of stable carbon isotopic values within the Tepe Yahya caprines, we must assess the differing ratios of C_3_ and C_4_ plants in animal diets. Bayesian mixing models are tools for investigating dietary choices and resource utilization, providing estimations of percentage of different food groups [91]. Mixing models have been used in a variety of cases, on human and canine populations, as well as, assessing weaning age of infants [92,93]. However, the usefulness of mixing models is contingent on the original input parameters, which are based on knowledge of the available plants and animals in the environment [94]. In this study, we use a hypothetical model to guide interpretations of overall caprine diets at the site. Using a generalized mixing model allows us to estimate the relative proportions of general C_3_ and C_4_ plants in the diet of caprines [95]. The percentage of C_3_ plants in the diet provides some environmental context to the expected abundance of C_3_ and C_4_ plants consumed without relying on knowledge of specific species or consumption patterns. A generalized model is preferable given our limited knowledge of the diversity of wild plants and the extent to which cereals were provided as fodder. Attempting to establish accurate input parameters would be tenuous at best.

Using the R package “simmr” as the basis for the mixing model [96], we modeled C_3_ plants as having a mean δ^13^C value of –22‰ and (standard deviation: 1.5‰) and C_4_ plants as having a mean δ^13^C value of –5.5‰ (standard deviation: 3.0‰), after Pearson [97] and O’Leary [98], S1 file. The model can then be used to calculate the percentage C_3_ of each specimen’s diet and average estimates for the three archaeological periods at Tepe Yahya (Neolithic/Chalcolithic, Proto-Elamite, and Bronze Age).

## Results

A total of 254 caprines in this assemblage produced acceptable isotope results, a 72% success rate of the 334 specimens sampled (Table 1). Fifty-three samples failed to produce enough collagen during extraction. Another 30 were removed after analysis for atomic C:N values outside the accepted range [88,99]. See S1 File for the full list of samples. The early periods, VII to V, produced the least amount of data, with only 22 successful samples. This is

**Table 1:** Table of samples in assemblage and successful isotope results.

	VII	VI	V	IVC	IVB	IVAB	IVA	Total
**Period total**	37	24	17	33	107	28	109	355
**Total attempted**	30	19	17	30	105	28	105	334
**Failed collagen collection**	23	14	3	8	3	0	2	53
**Outside accepted range**	1	0	3	7	12	1	6	30
**Total accepted**	6	5	11	18	90	27	97	254
**Percent Successful**	16%	21%	65%	55%	84%	96%	90%	72%

not surprising, given the low return of collagen-based ZooMS results for these same periods [46].

**Fig 4:**
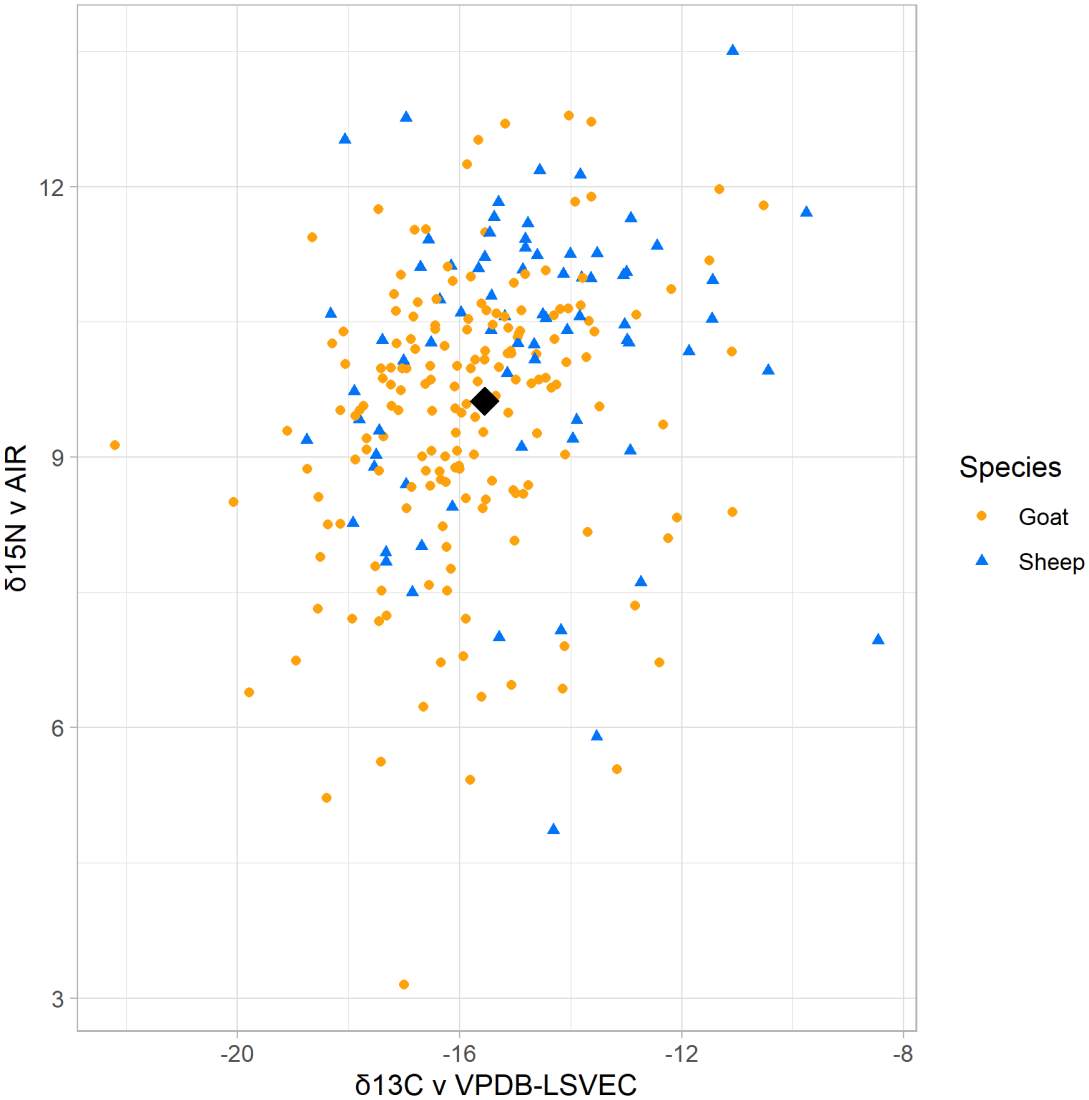
Plot of all Tepe Yahya Caprines isotope values. All isotope values in the assemblage plotted in a single graph. Goats are always depicted in orange, and sheep are always depicted in blue. The average is marked with a black diamond.

**Fig 5:**
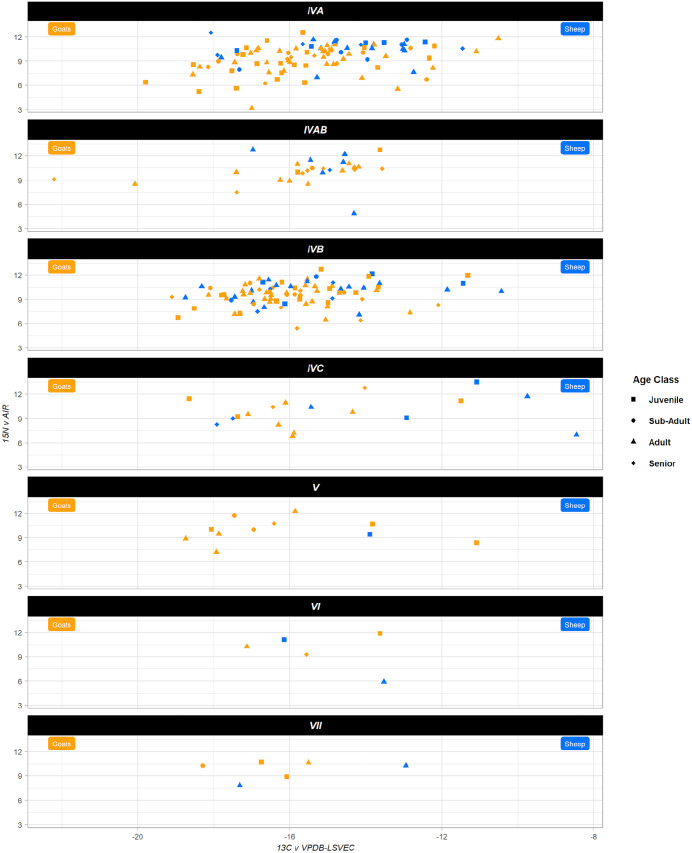
Plot Isotopic values of Tepe Yahya. Period VII, the earliest period is on the bottom, Period IVA, the latest, is at the top. Goats are in orange; sheep are in blue, and shapes are for the different age classes; see the legend.

**Fig 6:**
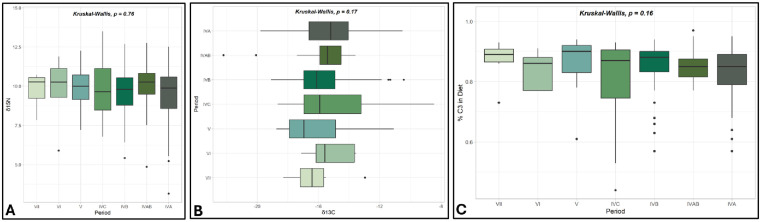
Boxplots for δ^15^N, δ^13^C, and percent C_3_ in diet by period. A) Boxplot of δ^15^N values, B) Boxplot of δ^13^C values, as typically presented along the X axis, C) Boxplot of the mean percent C_3_ in the diet.

The δ^13^C assemblage average is −15.6‰, standard deviation of 1.9, and the δ^15^N assemblage average is 9.6‰, standard deviation of 1.6 (Table 2, Fig 4). The range (Δ) of δ^13^C and δ^15^N values in both species is wide: the Δ^13^C is 13.7‰, and the Δ^15^N is 10.4‰ (Table 2). Fig 5 displays the data by period, highlighting the low number of samples in the early periods. Despite some drastic outliers in the assemblage (see below), the wide range within the isotopic values buffers the effect of any single outlier. These averages remain consistent throughout time. We conducted non-parametric Kruskal-Wallis tests to examine any statistical significance between periods because of the unequal sample sizes. We found no significant differences between periods for the δ^13^C values, δ^15^N values, or the percentage of C_3_ plants in the diet (Fig 6).

**Table 2:** Carbon and nitrogen isotope results for Tepe Yahya caprines separated by period.

	NISP	δ^13^C average	δ^13^C minimum	δ^13^C maximum	Δ^13^C	δ^15^N average	δ^15^N minimum	δ^15^N maximum	Δ^15^N	C:N_AT_ average
Period VII	6	−16.2	−18.3	−13.0	5.3	9.8	7.4	10.7	3.3	3.3
Period VI	5	−15.2	−17.3	−13.5	3.8	9.7	5.9	11.1	5.2	3.5
Period V	11	−16.2	−18.7	−11.0	7.7	9.9	7.2	12.3	5.0	3.3
Period IVC	18	−14.8	−17.9	−8.5	9.5	9.8	6.8	13.5	6.7	3.4
Period IVB	90	−15.8	−19.0	−11.3	7.7	9.6	6.5	12.7	6.2	3.4
Period IVAB	27	−15.7	−22.2	−13.6	8.6	10.0	4.9	12.7	8.1	3.3
Period IVA	97	−15.3	−18.6	−10.5	8.0	9.5	3.2	12.5	9.4	3.4
Total	254	−15.6	−22.2	−8.5	13.7	9.6	3.2	13.5	10.4	3.4

The species identifications for all the mandibles in the assemblage were previously established [46]. This is a goat-dominated assemblage, with 179 goats and 75 sheep (Table 3). Goats in the assemblage have an average δ^13^C of −15.8‰ and δ^15^N of 9.4‰. The average δ^13^C for sheep is −14.9‰ and 10.1‰ for δ^15^N. The two species have remarkably similar average isotopic values, with only a 1‰ difference between the δ^13^C averages of sheep and goats and a 0.6 ‰ difference in δ^15^N averages, just under a standard deviation for the assemblage. The overall homogeneity between sheep and goats suggests that the animals were not herded separately, rather, herds contained both sheep and goats.

**Table 3:** δ^13^C and δ^15^N results for Tepe Yahya sheep and goats by period.

	Goat NISP	Goat δ^13^C average	Goat δ^15^N average	Sheep NISP	Sheep δ^13^C average	Sheep δ^15^N average
Period VII	4	−16.7	10.1	2	−15.1	9.0
Period VI	3	−15.4	10.5	2	−14.8	8.5
Period V	10	−16.4	9.9	1	−13.9	9.4
Period IVC	11	−15.8	9.8	7	−13.3	9.9
Period IVB	63	−16.0	9.8	27	−15.4	10.0
Period IVAB	20	−15.9	10.0	7	−15.1	10.4
Period IVA	68	−15.6	9.0	29	−14.6	10.5
Total	179	−15.8	9.4	75	−14.9	10.2

The animals in the assemblage’s ages-at-death span from two months to over eight years old. The caprines were divided into four age groups: juvenile (under 12 months old), sub-adult (one to three years old), adult (three to eight years old), and senior animals (eight years and older), to examine if management patterns dictating diet were influenced by animal age (Table 4). Again, the values are remarkably homogeneous, clustering tight to the overall mean. Caprines appear to have been weaned early as juveniles do not consistently show elevated δ^15^N expected from nursing animals [69]. Only the senior group, animals eight years or older based on tooth wear, varies slightly from other age groups, with an δ^13^C average of −16.3‰, only 0.8‰ below the overall mean, and a δ^15^N average of 10.3‰, only 0.7‰ above the overall mean.

**Table 4:** δ^13^C and δ^15^N results for Tepe Yahya caprines organized by age group.

Group	Age range	NISP	δ^13^C average	Δ^13^C	δ^15^N average	Δ^15^N	Number Sheep: Goat
Juvenile	Under 12 months	64	−15.5	8.7	9.7	8.3	13:51
Sub Adult	1-3 years	72	−15.5	8.6	9.7	7.9	21:51
Adult	3-8 years	107	−15.6	11.6	9.4	9.1	38:69
Senior	8 years & older	11	−16.3	8.6	10.3	5.3	3:8

## Discussion

### General patterns

At Tepe Yahya, 84% of caprines display a mixed C_3_/C_4_ diet based on their isotope ratios. There is no strong patterning between sheep and goats indicating they were likely herded together, as is regularly observed past and present [37]. Poorer preservation of samples from earlier periods limits our understanding of them, but the mixed pattern is consistent through time. People at Tepe Yahya utilized more goats than sheep every period, averaging one sheep for every two goats [46]. Goats are better than sheep at extracting water from the plants they consume during digestion, making them better suited for survival in arid environments [100]. By investing more in goats, people could worry less about the impact of longer durations of aridity.

The mixed diet signal prevails between age groups, representing short-term and longer-term dietary input. However, within this mixed C_3_/C_4_ diet, there is a wide range of values. The δ^13^C values span −22‰ to −8‰, and δ^15^N values 4‰ to 13.5‰. Thus, we are likely observing multiple, mixed herds utilizing the landscape in different ways, with varying access to plants, be it seasonally or by geographic area.

The utilization of animals at all ages obscures direct indications of specialized caprine use at Tepe Yahya. However, the early weaning of juveniles observed in the assemblage may suggest increased desire for milk from older animals, while the younger ones were culled for meat [62]. Sheep in periods IVB and IVA lived to older ages, potentially demonstrating an increase in utilization of these animals for textiles, as is seen throughout the Bronze Age in southwestern Asia [46]. We do not observe dietary patterns suggestive of preferential or specialized feeding for animals for different purposes, such as the old sheep desired for wool. Only further avenues of delineation may reveal specifics in the generally wide dietary breadth of the Tepe Yahya caprines.

The Tepe Yahya caprines have variable δ^15^N values; however, the average remains steady at 9.5‰. This can be considered generally elevated compared to what we might expect from herbivores [101]. Similarly, elevated nitrogen values are common in Northern Iranian faunal and human remains, suggesting an overall aridity effect in Iranian δ^15^N values [68,102]. As aridity increases, soils lose nitrogen more rapidly, enriching the plant tissues and subsequent animal tissues [103,104]. Finding higher nitrogen values in a semiarid valley, such as where Tepe Yahya sits, is not unexpected.

### Percentage of dietary input

The isotopic mixing model shows that the diversity in δ^13^C values translates into stark differences between individuals in dietary composition, with percent C_3_ plants ranging from 44−97%. The lowest δ^13^C value in the assemblage is from a goat that had a diet of 97% C_3_ plants. The highest δ^13^C value comes from a sheep, which had only 44% dietary input from C_3_ plants. Caprines with δ^13^C values around the average, −15.5‰, consumed a diet of 85% C_3_ plants, according to our model. Table 5 details the percentage of C_3_ in the diet based on age and species, then grouped by period.

**Table 5:** Percent C_3_ dietary input for the Tepe Yahya caprines. A) C_3_ dietary input delineated by species and age, B-D) Period average C_3_ dietary input, then delineation by species and age.

A) Assemblage Goats			Assemblage Sheep		
	% C_3_	NISP		% C_3_	NISP
juvenile goats	85%	51	juvenile sheep	79%	13
sub-adult goats	86%	21	sub-adult sheep	82%	8
adult goats	86%	82	adult sheep	81%	42
senior goats	86%	25	senior sheep	86%	12
**Total goats**	**86%**	**179**	**Total sheep**	**82%**	**75**
**B) Early Periods**			**Average: 85% C3 in diet**		
	**% C**_**3**_	**NISP**		**% C**_**3**_	**NISP**
juvenile goats	81%	6	juvenile sheep	84%	2
sub-adult goats	91%	2	sub-adult sheep	–	–
adult goats	90%	6	adult sheep	80%	3
senior goats	88%	2	senior sheep	–	–
**Total goats**	**88%**	**16**	**Total sheep**	**82%**	**5**
**C) Proto-Elamite (Early Bronze)**			**Average: 80% C3 in diet**		
	**% C**_**3**_	**NISP**		**% C**_**3**_	**NISP**
juvenile goats	83%	3	juvenile sheep	67%	2
sub-adult goats	–	–	sub-adult sheep	–	–
adult goats	87%	6	adult sheep	61%	3
senior goats	84%	2	senior sheep	92%	2
**Total goats**	**85%**	**11**	**Total sheep**	**73%**	**7**
**D) Bronze Age**			**Average: 85% C3 in diet**		
	**% C**_**3**_	**NISP**		**% C**_**3**_	**NISP**
juvenile goats	86%	42	juvenile sheep	80%	9
sub-adult goats	86%	19	sub-adult sheep	82%	8
adult goats	85%	70	adult sheep	83%	36
senior goats	86%	21	senior sheep	85%	10
**Total goats**	**86%**	**152**	**Total sheep**	**83%**	**63**

**Fig 7:**
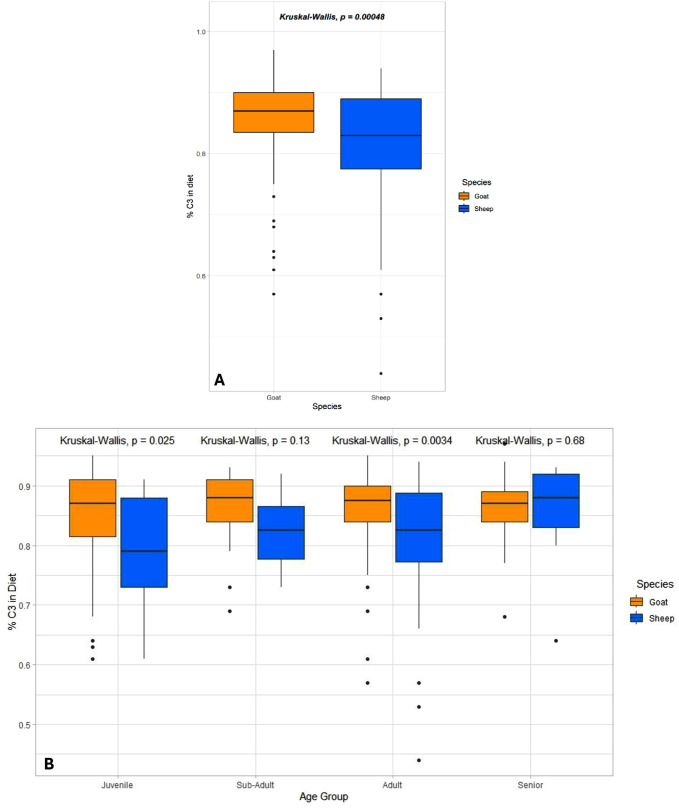
Boxplot of percent C_3_ in the diets of goats and sheep and goats and sheep by age. A) Kruskal-Wallice p-value is < 0.05 (p = 0.00048) indiciating signficant differerences in C_3_ consumpition of sheep and goats. B) Boxplot displaying percent of C_3_ in diet by age group and species. Kruskal-Wallice p-values for Juvenile (p = 0.025) and Adult (p = 0.0034) sheep and goats is significantly different.

Overall, the averages of % C_3_ in the diet of Tepe Yahya caprines remained remarkably stable over time (Fig 6C, above). These results indicate that while C_4_ plants, likely grasses, were readily accessible, caprine diets were primarily composed of C_3_ plants, the domesticated crops or wild C_3_ shrubs on the landscape. However, a Kruskal-Wallis test reveals that the percent of C_3_ in the diet between goats and sheep is statistically significant, p < 0.05 (p = 0.00048) (Fig 7A). Additionally, when examined by species and age group, the juvenile sheep and goats (p = 0.025) and the adult sheep and goats (p = 0.0034) had statistically significant differences between the species (Fig 7B). Goats at Tepe Yahya tended to consume more C_3_ plants than sheep, particularly for juvenile and adult animals. This might suggest that prime-age sheep were kept in a way that allowed them more access to C_4_ plants than their goat counterparts.

We do not observe a correlation in diet or percentage of C_3_ plants with the climatic conditions of the region (outlined above). The average of C_3_ does not vary enough to suggest environmental shifts towards more arid-adapted C_4_ plants. The range of δ^15^N values also remains wide over time, giving no indication of increased environmental aridity affecting any specific period. Thus, the differences in C_3_ consumption between sheep and goats more likely stems from animal preference or human interference than it is from climatic pressure on the environment.

### Dietary outliers

The isotopic values of a single or small set of caprines do not represent all caprine diets for a given site. Previous work estimates that most variability within a given caprine herd is captured by sampling 40 individuals per group [105]. Many zooarchaeological isotopic datasets do not meet the 40-sample criterion because of variable preservation. Thus, it is challenging to determine true dietary outliers from expected variation. Outliers can be defined as falling more than two standard deviations from the mean outside the 95% confidence intervals. In statistics, outliers are sometimes thrown out to preserve data integrity [106]. However, outliers in archaeological assemblages can be meaningful because of the human-driven component. The outliers serve as windows into actions outside of normal conditions or expectations. Knowing that most animals consumed a mixed C_3_/C_4_ diet throughout Tepe Yahya’s occupation highlights the animals in the same assemblage with a different diet. Since the environment provided a mix of C_3_ and C_4_ plants, the deviation from this mixed dietary signal implies a level of constraint for these animals [107]. Animals with outlying dietary signatures are visible in the large dataset (Fig 4). Forty-six animals, or 18% of Tepe Yahya sheep and goats, are dietary outliers (Table 6).

**Table 6:** Animals identified isotopically as outliers based on δ^13^C and δ^15^N values or, in a few cases, both. The outlying values for each sample are colored, see the key at the bottom.

ID	Outlier for	δ^13^C	δ^15^N	Species
93	Nitrogen	−13.5	**5.9**	Sheep
105	Carbon	***−11.1***	8.39	Goat
116	Carbon	**−18.7**	8.87	Goat
121	Both	***−11.1***	***13.5***	Sheep
126	Carbon	***−9.8***	11.71	Sheep
127	Carbon	***−8.5***	6.97	Sheep
131	Nitrogen	−14.0	***12.79***	Goat
137	Carbon	**−18.7**	11.44	Goat
143	Carbon	−11.5	11.18	Goat
155	Nitrogen	−14.1	**6.43**	Goat
159	Carbon	**−18.9**	6.74	Goat
166	Carbon	***−11.4***	10.96	Sheep
170	Carbon	**−18.5**	7.89	Goat
174	Nitrogen	−15.1	**6.47**	Goat
190	Nitrogen	−15.2	***12.7***	Goat
200	Carbon	**−19.1**	9.29	Goat
204	Carbon	***−10.4***	9.96	Sheep
208	Carbon	***−11.3***	11.97	Goat
215	Carbon	**−18.3**	10.59	Sheep
221	Nitrogen	−15.6	**6.34**	Goat
229	Carbon	**−18.8**	9.19	Sheep
248	Carbon	**−20.1**	8.5	Goat
250	Carbon	**−22.2**	9.13	Goat
277	Carbon	**−18.4**	8.25	Goat
278	Carbon	−11.5	10.53	Sheep
279	Carbon	**−18.6**	7.32	Goat
286	Carbon	**−18.2**	8.26	Goat
307	Carbon	***−11.1***	10.17	Goat
313	Carbon	**−18.3**	10.26	Goat
315	Carbon	***−10.5***	11.79	Goat
319	Carbon	**−18.1**	10.03	Goat
330	Carbon	***−11.9***	10.17	Sheep
331	Carbon	**−18.1**	10.39	Goat
336	Carbon	**−18.1**	9.52	Goat
337	Nitrogen	−13.6	***12.72***	Goat
343	Nitrogen	−17.0	***12.76***	Sheep
346	Nitrogen	−14.3	**4.86**	Sheep
370	Nitrogen	−15.8	**5.42**	Goat
377	Carbon	**−18.1**	12.52	Sheep
379	Nitrogen	−13.2	**5.54**	Goat
380	Nitrogen	−17.0	**3.15**	Goat
382	Both	**−19.8**	**6.39**	Goat
384	Both	**−18.4**	**5.22**	Goat
390	Carbon	**−18.5**	8.56	Goat
391	Nitrogen	−16.7	**6.23**	Goat
400	Nitrogen	−17.4	**5.62**	Goat
Key	**More negative carbon**	***More positive carbon***	**Lower nitrogen**	***Higher nitrogen***

#### Carbon outliers.

The average δ^13^C for the assemblage is −15.5‰. Isotopic values lower than −18‰ indicate a predominantly C_3_ diet. Conversely, when δ^13^C is above −12‰, diets are comprised of predominantly C_4_ plants [56]. Twenty individuals (17 goats, and three sheep) have δ^13^C less than −18‰. This means these animals are consuming almost exclusively C_3_ plants. Goat 250 has a δ^13^C of −22.5‰, the lowest value in the assemblage. Our mixing model shows this goat’s diet was 97% C_3_ plants.

Substantially more goats than sheep consumed predominantly C_3_ diets, highlighting one avenue of potential species-specific differences in management. One possibility is that these animals were kept closer to or inside the settlement and possibly foddered, preventing the ability to consume wild C_4_ plants. Domestic crops of wheat, barley, or oat were the likely C_3_ fodder sources. However, goats are browsers that tend to prefer a variety of woody shrubs [100]. They are more independent than sheep and selective about the foods they consume [108]. Thus, a second possibility is that these goats had increased access to abundant forge of their choosing, seeking out their preferred foods, which happened to be C_3_ plants. More work is needed to confirm either possibility.

The three sheep with a predominantly C_3_ diet must have been kept closer, if not within, the settlement and foddered with the domesticated crops, wheat or barley. Two of these three sheep have evidence of carnivore gnawing, indicating the accessibility of these remains to dogs in and around Tepe Yahya.

Twelve individuals, five goats, and seven sheep have a more positive δ^13^C values, greater than −12‰, indicating predominately C_4_ diets. Period IVC, c. 3300–2800 BCE, has one-third of all the predominant C_4_ consumers. Sheep 127 has the highest δ^13^C, −8.5‰, which, according to our mixing model, suggests only 44% C_3_ input and 56% C_4_ input. While in each period, some animals are being managed in a way that removes the opportunity to consume more C_3_ plants, this practice was more pronounced in Period IVC. Sheep are grazers and prefer low-lying grasses and other plants on the landscape [108]. If there were more C_4_ grasses around Tepe Yahya, this could account for the higher C_4_ input of sheep compared to goats. These sheep might have also had access to higher elevation pastures, which would also have more arid adapted plants [79,109]. Alternatively, it is possible that sheep were foddered with millet and goats were not. Unfortunately, C_4_ input from millet and wild plants are indistinguishable, and without other avenues of investigation, we cannot say whether millet foddering played a substantial role.

#### Nitrogen outliers.

Animals with δ^15^N values that deviate more than 3‰ from the average, 9.6‰, are considered outliers, as this signifies approximately a trophic level difference [110–112]. Seventeen animals have outlying nitrogen values, four sheep and 13 goats (Table 6).

The highest δ^15^N, 13.5‰, comes from Sheep 121, a juvenile between two and six months old. The high value likely reflects the trophic increase observed in nursing juveniles from excess nitrogen in milk produced by lactating mothers [69,113]. The elevated value suggests this young sheep was still nursing when it died. This young sheep also had an outlying δ^13^C value above −12‰. This elevated value might suggest this sheep was born in an environment where C_4_ plants dominated, reflecting its mother’s consumption of a diet higher in C_4_ plants. The δ^13^C value of this sheep can direct us towards investigations of caprine birthing environments, which are rarely considered because of the difficulty to observe.

**Fig 8:**
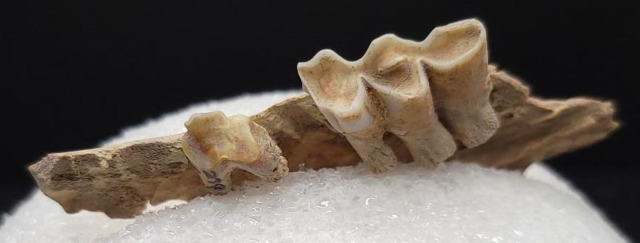
Pathological first molar of Goat 131. Teeth of Goat 131, with dental pathology of the first molar and heavy wear of the third molar.

The second highest δ^15^N value, 12.8‰, comes from Goat 131, who was at least eight years old, removing the weaning hypothesis as a possible explanation. Several possibilities could explain this elevated δ^15^N. The goat could have consumed exclusively fertilized crops enriched in nitrogen [70,71]. Another possibility is the δ^15^N value reflecting a period of prolonged stress at the end of the goat’s life. Scholars have observed increases in δ^15^N values of fish, human, and other mammalian tissues during periods of stress, injury, malnutrition, or disease [114–116]. The body enters catabolysis, utilizing its own nitrogen reserves [117]. The resulting δ^15^N for animals with pathologies may not accurately reflect the long-term diet [118]. This animal had a severe dental pathology, which may have contributed to elevated stress if it was causing pain and difficulty eating (Fig 8). The bone collagen of the pathological mandible fragment is more likely to reflect the elevated nitrogen values than post-cranial elements of the same animal. This goat’s δ^13^C value is −14‰. While this indicates a more consumption of C_4_ plants in the diet, it is still within the mixed C_3_/C_4_ range. This correlation cannot be seen as causation, and not all animals with prominent dental pathologies have an elevated δ^15^N.

Twelve animals, two sheep and 10 goats, have at least a tropic level depleted their δ^15^N values. One likely reason for this is consumption of legumes. Many legume species, such as vetches, belonging to genera *Vicia* and *Astragalus,* are endemic to Iran [119]. These plants are nitrogen fixers, meaning they rely on atmospheric nitrogen instead of soil nitrogen [120]. As a result, their nitrogen values are lower than typical plants, with δ^15^N values typically between −1‰ and 2‰ [120]. Animals consuming large portions of legumes, be they wild across the landscape or grown specifically as fodder, will have lower nitrogen values, reflecting the lower nitrogen content of the plant tissues. The larger number of goats with lower nitrogen values may relate to their preference to seek out C_3_ plants on the landscape, which would include most legume species, and potentially speaks abundance of legumes available [119].

Several animals are outliers in both δ^13^C and δ^15^N. While the elevated values of Sheep 121 are more readily interpretable, the others, particularly those with lower nitrogen values, are not. Goats 382 and 384, both between six and twelve months old, have low δ^13^C and δ^15^N values. The more negative carbon values suggest more C_3_ plant consumption. Though these animals are still young, the lack of elevated nitrogen indicates they were wholly weaned and no longer consuming milk when they died. Together, the values allude to the consumption of unfertilized C_3_ plants as opposed to fodder with domestic crops that were likely fertilized. On the other side of the spectrum, Sheep 127, four to six years old, has a less negative δ^13^C but a similarly low δ^15^N. This sheep was eating exclusively C_4_ plants, and those plants also lacked fertilization.

The dietary outliers in this herd demonstrate that people exerted differing levels of control over animal diets. While many were allowed to the variety of plants on the landscape, some were intentionally restricted, particularly from consuming C_4_ plants. Animals that are right at the cusps of the cutoff for outliers here indicate the broad spectrum of diet and managerial strategies at Tepe Yahya. Overall, there is no patterning within the outlying animals and their the δ^13^C and δ^15^N values. Yet their presence in the assemblage is a sign and reminder that other management regimes existed, and that caprine management is a dynamic practice.

## Limited interpretative ability

Despite the large number of samples in this study, we lack sufficient contextual information to fully evaluate the environmental conditions in which these caprines were managed. The high variability of values in the dataset precludes a straightforward interpretation. No sites near Tepe Yahya have isotopic data for comparison. There are currently no other carbon and nitrogen isotopic values from plants, humans, or other animals in the immediate region. Thus, we must be careful before making overarching assumptions and consider the necessary additional context for the most accurate view of the past. This study will benefit from other project on legacy faunal material from southeastern Iran and provide necessary context for developing the regional understanding. More data for the region fosters a greater regional understanding of the paleoenvironments of southeastern Iran.

### Broader comparison

The Tepe Yahya data can be compared with caprines in other regions to examine overall management strategies. Five sites in northwestern Iran and two in northeastern Iran have 29 domestic caprine isotopic results, in addition to data from other species, humans, cattle, wild animals, and dogs cf. [68,121]. The δ^13^C values of the caprines span −20.3‰ to −16.5‰. The highest value of these animals is 1‰ lower than the Tepe Yayha average, indicating that these caprines consumed mostly C_3_ plants. Several cattle at one Northeast Iran site display a substantial C_4_ dietary input, δ^13^C value of −9.94‰, in contrast to the two caprines with a primarily C_3_ diet from the same site [68]. This provides an example of how people practiced different management strategies between the two species and further displaying the mixed C_3_/C_4_ diet of the Tepe Yahya sheep and goats. Enamel carbonates of caprines from nine Indus sites in Northwest India indicate a mixed C_3_/C_4_ diet that correlates with the known mix of vegetation on the landscape [107]. The caprines at the Indus sites also consumed more varied diets than their cattle counterparts, who had more C_4_-based diets [107]. Looking further, at Tell Humeida, Syria, between 3700 and 3300 BCE, sheep had an increase in C_4_ in their diet and high δ^15^N [122]. The authors propose that the C_4_ input comes from millet, as the wild animals do not display a similar increased C_4_ signature or higher δ^15^N values.

### Future directions

More work is necessary to contextualize and interpret the stable isotope data from the Tepe Yahya caprines. Here, we identify several avenues for more targeted investigation into the environment and animal management in southeastern Iran. Each study mentioned in the previous section has data from other species at the site to contextualize the caprine results. Examining other animals in the Tepe Yahya assemblage is a priority for gaining complementary insights. Isotopic ratios from wild animals will reveal whether their diets are similar or different in composition to the domesticated caprines [122,123]. Cattle are another avenue of comparison because of their increased reliance on water [124]; they are more likely to be kept closer to stable water sources. Cattle carbon isotopic values would reveal if they were consuming the same wide variety of plants or whether their diets were narrower and more controlled.

Other avenues of investigation include isotopic analysis of plants. Ancient, carbonized seeds could provide insights into isotopic variation due to environmental and water conditions, as done in Syria and Cyprus cf. [51,122]. Modern botanical surveys would also improve our understanding of past environments. Regions with a high environmental and climate variability may contribute to high ranges and outliers in the resulting isotope values of different species [125]. Although the present flora cannot be directly ascribed to the past, a modern collection of plants for species identification would provide a sense of the possible plants, followed by stable isotopic analysis, which would provide a base to investigate. At present, the possibility of this work being carried out by Western scholars is limited. However, this could allow collaborations with Iranian scholars interested in paleobotany or paleoenvironments.

Further examination of these caprines can also elucidate our understanding of the environment and animal management. Serial sampling of second and third molars for carbon, oxygen, and strontium isotopic analysis can elucidate seasonal and long-distance movements of the caprines. The carbon and oxygen isotopes within a tooth will reveal if consumption of plants was seasonal, C_3_ in some parts of the year and C_4_ in others, which when combined with paleoenvironmental interpretations, indicate whether animals moved vertically between different elevations between seasons or remained local year-round. As a highland site 1500 masl, people herding caprines for those of Tepe Yahya likely utilized pastures at higher and lower elevations. Herders take advantage of the different bioavailability of plants that follow elevation gradients. This type of movement also serves to safeguard pastures from excessive overgrazing [126]. The strontium values will reveal if animals were born elsewhere and brought to Tepe Yahya at a later age, implying long-distance trade of animals throughout the Zagros Mountains and the Iranian Plateau. Future strontium analysis can reveal whether caprines were traveling further in the Bronze Age, when more animals from more herds appear to be supplying Tepe Yahya. New methods for obtaining nitrogen values from tooth enamel could continue to shed light on the environmental conditions [127].

## Conclusion

The dietary and management insights from carbon and nitrogen isotopes highlight that the Tepe Yahya caprines are still a valuable source of information. Herds of sheep and goats at Tepe Yahya were likely mixed and not limited to strict managerial regimes. Animals were pastured with a variety of access to the plants in the landscape. The diets of the caprines have a higher average carbon value compared to other sites in Iran. The high percentage of C_4_ plant consumption in most animals confirms the predicted semi-arid environment of the Sogun Valley and potentially other high-elevation valleys in the Zagros. This wide scale isotopic undertaking revealed the presence of animals with outlying ratios of carbon, nitrogen, or both that would be otherwise invisible with a smaller sample size. These outliers expose specific differences in management, conveying the diversity in possibilities of animal management within a single site and landscape.

Returning to museum collections promotes new avenues of research and highlights the value of continued investment in curation. These faunal materials still have value, both culturally and for knowledge production. Increasing the number of comparable faunal datasets benefits our understanding of the many varieties of animal management practices. The ancient people of southwestern Asia lived in many different environments and settlements, prioritizing caprines in multiple ways. Understanding animal management in one region does not dictate an understanding of another [128]. The data presented here complements their ongoing work documenting legacy faunal collections in Iran, France and Canada from Iranian archaeological contexts, while also contributing to the overall number of Iranian sites with faunal and isotopic information [15,121]. We hope this project marks the start of a series of future zooarchaeological and ethnobotanical studies in this region that will expand understanding of past lifeways in the Sogun region during the Neolithic and Bronze Age periods.

## Supporting information

S1 File Tepe Yahya Caprines Carbon and Nitrogen full results. Extended carbon and nitrogen stable isotopic information.
(XLSX)

## Abbreviations

NISP: Number of Identified Specimens
ZooMS: Zooarchaeology Mass Spectrometry
EA-IRMS: Elemental Analyzer Isotope Ratio Mass Spectrometer
